# Hypocholesterolemia is an independent risk factor for depression disorder and suicide attempt in Northern Mexican population

**DOI:** 10.1186/s12888-018-1596-z

**Published:** 2018-01-15

**Authors:** Marcela Segoviano-Mendoza, Manuel Cárdenas-de la Cruz, José Salas-Pacheco, Fernando Vázquez-Alaniz, Osmel La Llave-León, Francisco Castellanos-Juárez, Jazmín Méndez-Hernández, Marcelo Barraza-Salas, Ernesto Miranda-Morales, Oscar Arias-Carrión, Edna Méndez-Hernández

**Affiliations:** 1 Instituto de Investigación Científica, Universidad Juárez del Estado de Durango, Universidad S/N esquina Volantín Zona Centro CP 34000, Zip Code 34000 Av., Durango, Mexico; 2 Hospital General 450, Servicios de Salud de Salud, Zip Code 34206, Durango, México; 3 Departamento de Biotecnología, Universidad Autónoma Metropolitana, Ciudad de México, México Zip Code 09340,; 4 Facultad de Ciencias Químicas, Universidad Juárez del Estado de Durango, Zip Code 34000, Durango, México; 5 Unidad de Trastornos del Movimiento y Sueño (TMS), Hospital General Dr. Manuel Gea González, Zip Code 14080, Ciudad de México, México; 6 Subdirección de Investigación en Salud, Servicios de Salud de Durango, Zip Code 34000., Durango, México

**Keywords:** Suicide attempt, depression, cholesterol

## Abstract

**Background:**

Cholesterol has been associated as a risk factor for cardiovascular disease. Recently, however, there is growing evidence about crucial requirement of neuron membrane cholesterol in the organization and function of the 5-HT_1A_ serotonin receptor. For this, low cholesterol level has been reported to be associated with depression and suicidality. However there have been inconsistent reports about this finding and the exact relationship between these factors remains controversial. Therefore, we investigated the link between serum cholesterol and its fractions with depression disorder and suicide attempt in 467 adult subjects in Mexican mestizo population.

**Methods:**

Plasma levels of total cholesterol, triglycerides, and high-density lipoprotein cholesterol (HDL-c) and low density lipoprotein cholesterol (LDL-c) were determined in 261 MDD patients meeting the DSM-5 criteria for major depressive disorder (MDD), 59 of whom had undergone an episode of suicide attempt, and 206 healthy controls.

**Results:**

A significant decrease in total cholesterol, LDL-cholesterol, VLDL-cholesterol and triglyceride serum levels was observed in the groups of MDD patients and suicide attempt compared to those without suicidal behavior (*p* < 0.05). After adjusting for covariates, lower cholesterol levels were significantly associated with MDD (*OR* 4.229 *CI* 95% 2.555 – 7.000, *p<*.001) and suicide attempt (*OR* 5.540 *CI* 95% 2.825 – 10.866, *p<*.001)

**Conclusions:**

These results support the hypothesis that lower levels of cholesterol are associated with mood disorders like MDD and suicidal behavior. More mechanistic studies are needed to further explain this association.

## Background

Suicide is one of the most disastrous outcomes of psychiatric disorders [1]. It is a significant public health problem and is one of the leading causes of death worldwide [2–4]. In Mexico the rate of suicide is a current health problem that is accentuated by the fact that it is a country with an emerging market economy. In recent years, Mexico has presented an increase in its suicide rate. For example, between 2000 and 2014 there was an increase in the suicide rate from 3.5 to 5.2 cases per 100,000 inhabitants (http://bibliodigitalibd.senado.gob.mx/handle/123456789/3181) [5].

Suicidal behavior includes a wide spectrum of behaviors, such as completed suicides, high-lethality suicide attempts, and low-lethality suicide attempts [6, 7]. Although roughly 60% of all suicides occur in the context of depressive disorders [7, 8] it is still challenging for clinicians to predict suicide risk in patients with depression. For this reason, increased attention has been paid to potential biomarkers for suicide in patients with major depressive disorder (MDD) and suicidal behavior [1, 9].

Many studies have confirmed that biological markers might be linked to suicidality, among which serum lipid levels might play an important role [2, 10–12]. However, there has been considerable controversy about the association between serum lipid levels and suicidality. Some human studies showed that suicide attempters had lower cholesterol levels [2, 11, 13, 14]others reported positive associations between cholesterol and completed suicide[2, 15–17]and some others indicated that there was no evidence for an association between serum cholesterol and suicidality [18, 19].Although the mechanism behind hypocholesterolemia and suicide has not been clearly defined, previous studies have suggested that altered cholesterol at synaptic lipid rafts may cause a reduction in serotonin communication [2].

In the Mexican population, very few studies have examined the association between serum lipid levels and the presence of depression and suicide attempt, and none have reported the frequency of hypocholesterolemia in depressed patients and suicide attempters. Here, we hypothesized that: 1) serum lipid levels are reduced in subjects with depression and suicide attempt; 2) Hypocholesterolemia is a risk factor associated with depression and suicide attempt.

## Methods

### Study population

After the approval of protocol by Research and Ethics Committee of the General Hospital 450 (No. 12/0306-2015), a case-control study was conducted with 261 MDD adult patients, 59 of these also had a recent episode of suicide attempt and 206 healthy adult volunteer controls of both genders; all subjects provided informed consent. Study subjects were recruited from the Mental Health Hospital “Dr. Miguel Valle Bueno” (Secretary of Health) and psychiatry service of the General Hospital 450 (Secretary of Health) and General Hospital “Dr. Santiago Ramón y Cajal” (ISSSTE), in Durango City, Mexico, from June2015to December 2016.

MDD diagnosis was made by trained psychiatrists according to DSM-5 criteria. We defined suicide attempt as "a non-fatal, self-directed, potentially injurious behavior with an intent to die as a result of the behavior" as defined by the Center for Disease Control and Prevention (https://www.cdc.gov/violenceprevention/suicide/definitions.html) [20]. For our recruitment, we considered only participants that required hospitalization.Subjects in the case group were matched with subjects in the control group based on age, sex and body mass index (BMI).The latter, in order to exclude nutritional state as a confounding factor and due to previous association between BMI and cholesterol levels. [21]. Control subjects were apparently healthy persons without any symptoms or signs of MDD based on a clinical examination at inclusion. Use of lipid lowering drugs, such as statins was considered a confounding factor due to the diverse effects it may have on cellular mediation of inflammation and immunity [22] in conjunction with its known effect on depression [23] and was, therefore, an exclusion criterion. Lastly, we excluded those with chronic diseases (hepatic disorders, diabetes mellitus, hypertension, cardiovascular disease) due to their association with dyslipidemia. [24, 25]

### Lipid profile

Blood samples were obtained by venous puncture of all participants after an overnight fasting period. The lipid profiles were determined using the A15 Clinical Chemistry and Turbidimetry Systems (BioSystems) according to the manufacturer’s instructions. The level of serum lipids was evaluated based on total cholesterol (TC), high-density lipoprotein cholesterol (HDL-C), low-density lipoprotein cholesterol (LDL-C) and triglycerides (TG).Hypocholesterolemia was defined as a total cholesterol concentration of less than 150 mg/dl.

### Statistical Analyses

Numerical data were presented as mean±standard deviation (*SD*). Categorical variables were presented as proportions. Differences between the two case groups (MDD and MDD associated with suicide attempt) and control group (healthy subjects) were estimated using ANOVA (Kruskall Wallis test for skewed data) for numerical variables with an additional Bonferroni post-hoc test, and the chi-squared test for categorical variables. Additionally, our analysis was stratified by sex and logistic regression analysis was performed to determine the association between hypocholesterolemia (independent variable) and the presence of depression and suicide attempt (dependent variables). Values of *p*<0.05 were considered statistically significant. The Statistical Package for the Social Sciences (SPSS) for Windows version 21.0 (SPSS Inc., Chicago, IL) was used for data management and statistical analysis. Odds ratio (*OR*) and 95% confidence interval (95%*CI*) were determined, while *p* value <0.05 defined the statistical significance.

## Results

Clinical characteristics and laboratory parameters of the 467 enrolled subjects are shown in Table 1. The comparative analysis of the serum lipid levels between the study groups showed significant differences in the total cholesterol, LDL-cholesterol, VLDL-cholesterol, triglycerides serum levels and, hypocholesterolemia frequency (*p*<0.05). No significant difference in HDL levels were observed between groups.

**Table 1:** Mean cholesterol levels and hypocholesterolemia frequency according to mental impairment categories

	Healthy subjects	MDD	MDD associated w/suicide attempt	*P* value
n	206	202	59	--
Age, years	36.8 ± 6.6	37.3 ± 10.0	35.2 ± 10.5	0.363
Females / Males, n (%)	166 (80.5) /40 (19.5)	169 (83.7) /35 (16.3)	42 (71.2) /17 (28.8)	0.139
Obesity frequency (IMC>30 kg/m^2^), n (%)	55 (26.8)	63 (32)	15 (25.4)	0.431
Total cholesterol serum level, mg/dl	172.5 ± 25.3	167.9± 45.1	152.2 ± 39.0	*<*.001^‡,*,†^
HDL cholesterol serum level, mg/dl	46.0 ± 11.6	43.8 ± 14.5	46.0± 18.7	0.198
LDL cholesterol serum level, mg/dl	96.7 ± 29.4	84.0 ± 39.5	76.9 ± 32.5	0.014^*^
VLDL cholesterol serum level, mg/dl	28.9± 14.8	41.2± 23.0	37.6± 21.3	*<*.001^‡,†^
Triglyceridesserum level, mg/dl	142.7 ± 87.1	208.3± 119.7	172.8 ± 88.1	*<*.001^‡,*,†^
Hypocholesterolemia frequency (<150 mg/dl), n (%)	28 (13.6)	79 (38.2)	29 (49.2)	*<*.001

*MDD* Major depression disorder

Values are mean ± standard deviation

*Statistically significant difference between Healthy subjects and MDD associated with suicide attempt

^†^Statistically significant difference between MDD and MDD associated with suicide attempt

^‡^Statistically significant difference between Healthy subjects and MDD

Post-hoc analysis between the two groups comprised of patients with MDD with suicide attempt (*p<*0.001) and MDD only (*p<*0.001) showed significantly lower total cholesterol levels compared to the control group. Also, patients with MDD who were associated with suicide attempt had significantly lower total cholesterol levels compared with MDD only (*p* 0.016).

LDL levels were lower in the MDD associated with suicide attempt group with respect to the control group (*p* 0.013). Subjects with depression presented higher VLDL (*p<*0*.*001) and triglyceride levels (*p<*0*.*001) compared with healthy controls. Similarly, subjects with depression presented higher VLDL (p 0.043) and triglyceride levels (*p* 0.039) compared with MDD with suicide attempt. The frequency of hypocholesterolemia was significantly higher in the MDD and MDD with suicide attempt groups compared with the control group (*p<*0*.*001).

A subgroup analysis by gender was performed in order to separately assess the magnitude of the association between lipid serum levels in men and women with MDD and suicide attempt (Table 2). Both, men and women showed significantly lower total cholesterol levels in subjects with MDD associated with suicide attempt compared with the control group (*p*<0.05). However, only in women, did we observe a significant difference between the MDD-only group versus the control group. No such relationship was seen in males. The comparative analysis of the hypocholesterolemia frequency between the study groups showed significant differences in both genders (*p<*0.05).

**Table 2:** Analysis by gender of clinical characteristics and laboratory parameters

	Male			*p* value	Female			*p* value
	Healthy subjects	MDD	MDD associated w/suicide attempt		Healthy subjects	MDD	MDD associated w/suicide attempt	
n	40	36	17		166	171	42	
Age, years	38.1 ± 6.1	35.8 ± 10.5	37.3 ± 11.9	*0.592*	36.5 ± 6.7	37.5 ± 10.0	34.3 ± 9.9	*0.133*
Obesity frequency (IMC>30 kg/m^2^), n (%)	10 (25)	6 (17.1)	3 (17.6)	*0.664*	45 (27.3)	57 (34.8)	12 (28.6)	*0.322*
Total cholesterol serum level, mg/dl	182.5 ± 42.5	164.8 ± 48.6	140.2 ± 32.1	*0.002* ^‡,*^	170.1 ± 18.4	168.5 ± 44.4	157.1 ± 40.8	0.017*
HDL cholesterol serum level, mg/dl	46.4 ± 14.5	41.7 ± 15.8	44.1 ± 12.4	*0.566*	45.8 ± 10.2	44.2 ± 14.2	46.8 ± 20.9	*0.301*
LDL cholesterol serum level, mg/dl	100.8 ± 35.9	71.2 ± 37.2	71.7 ± 24.5	*0.011* ^‡^	94.8 ± 26.2	86.7 ± 39.5	78.9 ± 35.3	*0.165*
VLDL cholesterol serum level, mg/dl	30.9 ± 17.0	55.4 ± 33.4	29.8 ± 10.1	*0.002* ^‡,†^	28.1 ± 13.9	38.1 ± 18.9	34.9 ± 18.7	*0.004* ^‡^
Triglycerides serum level, mg/dl	137.7 ± 66.9	277.1 ± 167.2	157.6 ± 60.2	*<*.001^‡,†^	143.9 ± 91.4	193.7 ± 101.8	179.0 ± 97.2	*<*.001^‡,^*
Hypocholesterolemia frequency (<150 mg/dl), n (%)	8 (20)	17 (47.2)	9 (52.9)	*0.015*	20 (12)	62 (36.3)	20 (47.6)	*<*.001

Values are mean ± standard deviation

*Statistically significant difference between Healthy subjects and MDD associated with suicide attempt

†Statistically significant difference between MDD and MDD associated with suicide attempt

‡Statistically significant difference between Healthy subjects and MDD

A bivariate logistic regression using data matched on age, sex, and BMI showed a statistically significant association between hypocholesterolemia and MDD (*OR* 4.229 *CI* 95% 2.555 – 7.000, *p<* 0*.*001). In the same way, a statistically significant association between hypocholesterolemia and suicide attempt was observed (*OR* 5.540 *CI* 95% 2.825 – 10.866, *p<*0*.*001). Additionally, triglycerides were analyzed and we found the following Odds Ratios and 95% Confidence Intervals: for hypertriglyceridemia in MDD [3.528 (2.326-5.352); *p<*0*.*001]; for hypertriglyceridemia in MDD with suicide attempt [2.626 (1.411-4.885); *p=*0.002]*.* Upon further analysis, no significant association in the other variables.

## Discussion

Here, we show that lower serum cholesterol levels are linked with MDD and suicide attempt. Age and sex-adjusted analyses showed a clear association between serum cholesterol levels and the risk of depression and suicide attempt.

Previously, some studies have also shown an association between low cholesterol and increased risk of death due to injuries or suicide [2, 9, 26–29], but not in other reports [1, 30–33], even elevated cholesterol levels have been associated with suicide mortality in other studies [15, 33].

In Mexican subjects, there is only one research paper that has examined the possible link with hypocholesterolemia and suicide attempt in subjects with depression [34]. This study found no difference in lipid profiles between patients who had attempted suicide and those who had not. However, these authors studied only 33 patients with a major depressive episode (moderate to severe) comparing 18 subjects who had attempted suicide versus subjects who had never attempted suicide.

Although there is evidence of a link between low serum cholesterol levels and suicide in patients with depression [35, 36], the mechanism that may link serum lipids with suicidality is still unclear. It has been established that nearly all brain cholesterol is produced *in situ* through de novo synthesis and that adequate prevention of its uptake from the bloodstream is provided by selectivity of the blood-brain barrier [37–39]. Nonetheless, it is viable that decreased peripheral cholesterol in those individuals with psychiatric disorders occurs concurrently with cholesterol modifications that take place in distinct synaptic lipid rafts in neurons (by a common regulatory mechanism). This could produce the minimized activity of serotonergic communication and, consequently, give rise to instinctive responses and violent suicidal behavior [2, 40].

Cholesterol is the paramount constituent of cellular membranes in higher eukaryotes and is essential in membrane function and organization as well as dynamics and sorting. It is commonly found dispersed in a non-random form in specific areas (domains) in both biological and model membranes [41–43]. These areas, often denominated as ‘lipid rafts’ [43, 44], are thought to be fundamental in the preservation of the structure and function of the membrane. However, describing the spatiotemporal resolution of these domains has turned out to be a difficult task [43, 45]. It has been suggested that these formations be membrane domains in which signaling from a neurotransmitter may arise via a group of receptors, such as serotonin_1A_ (5-HT_1A_) receptor [46].

Previous studies demonstrated the imperative necessity of membrane cholesterol in the function and organization of the 5-HT_1A_ receptor [45, 47–52]. Results from additional studies showed that the fluidity of lipids considerably regulates the binding of serotonin (5-HT) in murine brain membranes. It is therefore expected that decreased levels of cholesterol would increase the fluidity of the cellular membrane. While, at the same time, minimal exposure of the 5-HT receptors would be found in the synaptic cleft [2, 53].

Reportedly, disturbance of rafts by cholesterol deficiency notably lowers agonist binding and coupling of G protein to 5-hydroxytryptamine 1A (5-HT1A) serotonin receptors in bovine hippocampal membranes [46, 47]. Serotonin_1A_ receptors typify one of the most formidable, evolutionarily primitive, yet largely conserved families of seven transmembrane *G* protein*-*coupled receptors (GPCRs) that span the membrane [45, 54]. Also, serotonergic signaling constitutes an important part in the formation and regulation of a multitude of functions such as behavioral, cognitive, and developmental [45]**.** Moreover, studies have demonstrated that there is an association between decreased 5-HT activity and suicide [2, 55].

It is noteworthy to mention that recent studies described crystal structures of GPCRs, including serotonin_1A_ receptor, that demonstrated structural proof of cholesterol binding sites [45, 56, 57].Currently, two conceivable pathways have been proposed by which membrane cholesterol could affect the structure and function of GPCRs: (i) by way of a direct/specific interaction with GPCRs, or (ii) via an indirect pathway by modifying the physical properties of the membrane in which the receptor is inserted, or as a result of an integration of both [45, 58].

About cholesterol levels and their relation to gender, our study showed that the decrease in total cholesterol levels occurred in both men and women. Other authors have reported a relationship between reduced cholesterol and suicidal tendencies only in males [13, 59–62]. However, it is worth noting that additional studies on the association between gender and serum cholesterol have been unconvincing.

A lack of consistency between different published reports coupled with the fact that, to date, it has not been possible to identify a cholesterol threshold level capable of precipitating a psychiatric disorder, suggests the presence of a non-linear relationship.

The existence of reports in which depression has been associated with increased cholesterol levels would support this hypothesis. A possible explanation for this, proposed the involvement of monoamine oxidase (MAO). The aforementioned model studies associated hypercholesterolemia with depression in hypercholesterolemic mice via monoaminergic metabolism. Specifically, they reported increased monoamine oxidase (MAO) A and B activity in the hippocampus of mice [63, 64]. Thus providing one possible reason why elevated levels of cholesterol are able to produce depression much like decreased levels are able to, but via independent mechanisms.

Besides total cholesterol, other studies that investigated the link between triglycerides, HDL cholesterol and LDL cholesterol, observed contrasting results between different populations. Our results showed higher levels of triglycerides in subjects with MDD and MDD associated with suicide attempt. These findings do not coincide with data previously reported in 2015 that observed decreased levels of triglycerides in subjects with suicidal attempts [65]. However, other authors report conflicting findings, even suggesting a positive association between triglyceride levels and the risk of suicidal behavior [15]. Considering LDL cholesterol levels, our results coincide with the conclusions of a meta-analysis published in 2016, which included a total of 36 different studies and found overall association between lower LDL levels and depression [66]. With respect to VLDL levels, which were significantly higher in MDD versus healthy controls, it is worth noting that few studies report VLDL levels. Taking this into consideration, our result is different from the result reported in a previous study [67] that showed significantly lower levels while our results demonstrate higher levels. With respect to HDL cholesterol levels, our results did not demonstrate significant differences when comparing subjects with MDD, subjects with MDD associated with suicide attempt and the control group. This fact contrasts with previous studies in which significant differences in HDL cholesterol between subjects with attempted suicide and healthy controls were shown [59, 68].

Lastly, we found a significant association between elevated triglyceride levels versus MDD and MDD with suicide attempt. Our finding coincides with a previous study which found a correlation between depressive symptoms and triglyceride levels [69] and suggestions by others which postulate that high triglyceride levels are associated with Type A personality traits, such as hostility, anger and domineering attitudes [70].

Several limitations of this study deserve to be mentioned. First, because our study is based on a case-control design, temporality could be not inferred with certainty. Whether hypocholesterolemia is a risk factor for developing depression and suicide attempt or merely an associated epiphenomenon can not be assured with certainty; second, we did not measure 24 S-hydroxycholesterol levels, which is a peripheral biomarker of brain cholesterol metabolism. However, it is expected that reduction of total cholesterol would reduce 24 S-hydroxycholesterol [71]. Besides, we did not evaluate dietary intake; however, because subjects in the groups of the study were enrolled from the same socio-cultural and economic background, it is expected that customary diets were similarly distributed. Finally, an additional limitation of our study is that we did not analyze the cardiovascular risk associated with serum cholesterol concentrations. In the future, it will be important to profoundly analyze the contradictory results reported with regard to cholesterol's role in depression. [63, 64]. This would help to verify if cholesterol is, in fact, a viable biomarker for neuropsychiatric disorders. It is evident that these results may be extrapolated only to a population that is similar to our own with similar exclusion criteria. Strengths of our study include the inclusion of incident cases of suicide attempt, which is a recognized tool to minimize analysis bias in the cross-sectional studies; also, the exclusion of individuals with lipid-lowering drugs allows us to control the potential source of bias.

## Conclusions

In conclusion, our results show that hypocholesterolemia is independently associated with depression and suicide attempt in adults of the Mexican population .This finding, if consistent in more studies in our population, could influence public health policies focused in the prevention of mental health disorders. Measuring cholesterol levels could be a minimally invasive, inexpensive and simple way to predict suicide risk in our population. The use of cholesterol levels as a biomarker would permit clinicians to efficiently obtain a laboratory result that, once combined with clinical evaluations and symptoms, could permit a more timely diagnosis. Additionally, it would be necessary to calculate the sensibility and specificity of this test in our population.

## Abbreviations

CHOL: Cholesterol
HDL: High-density lipoprotein
LDL: Low-density lipoprotein
MDD: Major depressive disorder
VLDL: Very low-density lipoprotein
